# Supplementary material for: Hybrid soliton dynamics in liquid-core fibres
Source: Nat Commun. 2017 Jun 29;8:42. doi: 10.1038/s41467-017-00033-5 (PMC5491526; doi:10.1038/s41467-017-00033-5)
Supplement: Supplementary file 1 — Supplementary Information [file 41467_2017_33_MOESM1_ESM.pdf]

File Name: Supplementary Information
Description: Supplementary Notes, Supplementary Figures, Supplementary Table and Supplementary References.

File Name: Peer Review file
Description:

Supplementary Note 1: Reinvestigation of Refractive Index Dispersion of Carbon Disulfide

Carbon-disulfide (CS_2) shows distinct electronic absorption in the ultraviolet and a strong absorption feature at 6.57 µm resulting from an anti-symmetric stretching mode [1]. As observed by Pfund [1], and Wilhelmi [2], and Ghosal *et al* [3], the infrared absorption band strongly influences the refractive index when approaching this resonance from the short-wavelength side (Suppl. Fig. 1). We fitted a two-term Sellmeier equation to the data of different groups (solid black line in Suppl. Fig. 1) from 300 nm to 5.8 µm with very high accuracy (R-square: 0.9994). Note that the main difference to the commonly used CS_2-single oscillator models (blue and green curves in Suppl. Fig. 1) emerge for wavelength bigger than 2 µm, which makes our dispersion model particularly valuable for the mid-infrared wavelength regime.

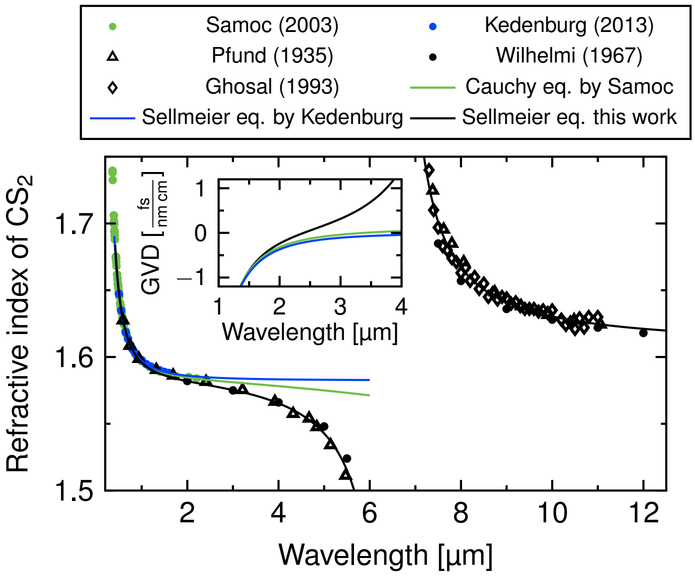

Supplementary Fig. 1: *Refractive index fit and model comparison. Experimental data points of the refractive index of bulk neat carbon disulfide measured by different groups. The solid black line refers to the fit obtained when using the two-oscillator Sellmeier equation (Eq. 1 in the main paper), whereas the two colored lines are calculated from either a one-oscillator model or a Cauchy equation (green: Samoc [4], blue: Kedenburg [5]). The names in the legend refers to the first author of the respective reference: Pfund [1], Wilhelmi [2], and Ghosal [3].*

Supplementary Note 2: Impact of Model Uncertainties on Simulation Results

In Suppl. Fig. 2a we compare our model with the two other models using supercontinuum simulation (incl. loss) with identical input parameters (*460 fs sech^2 pulse, 7 nJ*) and realistic nonlinearity of CS_2 (85% molecular fraction, i.e. $f_m = 0.85$). The results reveal a non-negligible difference between bandwidth and fission length between the models, whereas our model is closest to the early supercontinuum onset and the narrow bandwidth measured in the experiment.

In Suppl. Fig. 2b we investigated the impact of small uncertainties of the dispersion on our system indicators (i.e. bandwidth and supercontinuum onset energy) by comparing the output spectrum for three different core diameters. The results show only weak variations in the spectral bandwidth and small, but notable, differences in the measurable fission length.

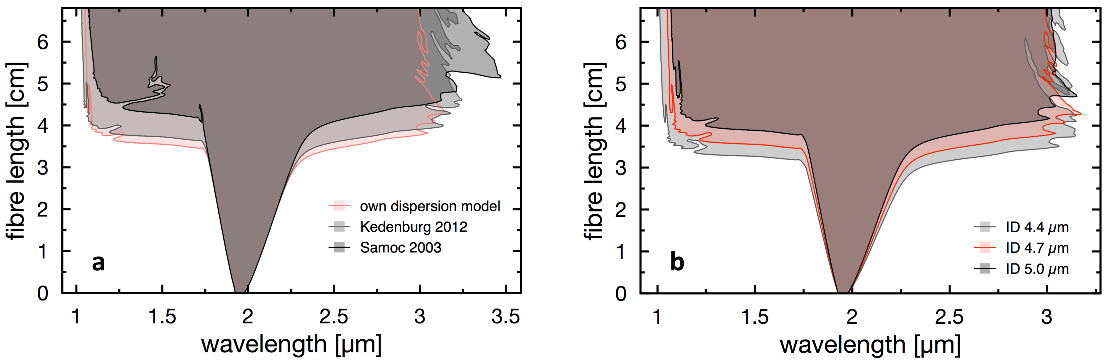

Supplementary Fig. 2: *Impact of dispersion on supercontinuum generation. Contour of simulated spectral evolution (at 40 dB contrast to the maximum total spectral intensity) of a 460 fs sech^2 pulse (7 nJ) in a CS_2-core for (a) a constant inner diameter of 4.7 µm but three different material dispersion models for CS_2 (Samoc et al. [4], Kedenburg et al. [5], and our model from the main paper) and for (b) our dispersion model for three different diameters.*

In Suppl. Fig. 3 we compare the simulated spectral evolution (including loss) for artificially varied molecular fraction f_m to demonstrate tit impact on spectral indictors of the new soliton dynamics, which are bandwidth, supercontinuum onset and noise susceptibility. The results show a significant bandwidth reduction and fission length increase for increasing f_m. The impact on the fission length increases for increasing f_m which demonstrates again the significance of the supercontinuum onset energy as an indicator for the soliton dynamics. Finally, the signature of the noise stability can be found in the spectral side lobes emerging around 1.4 µm and 2.7 µm at lower molecular fractions ($f_m \leq 0.7$), which indicate modulation instabilities as dominant broadening effect. Those signatures completely disappear for $f_m > 0.7$ and the broadening characteristic turns over to clean soliton fission and dispersive wave generation. Since modulation instabilities are dictated by photon noise, the absence of their spectral signatures indicates a much reduced impact of noise for increasing f_m.

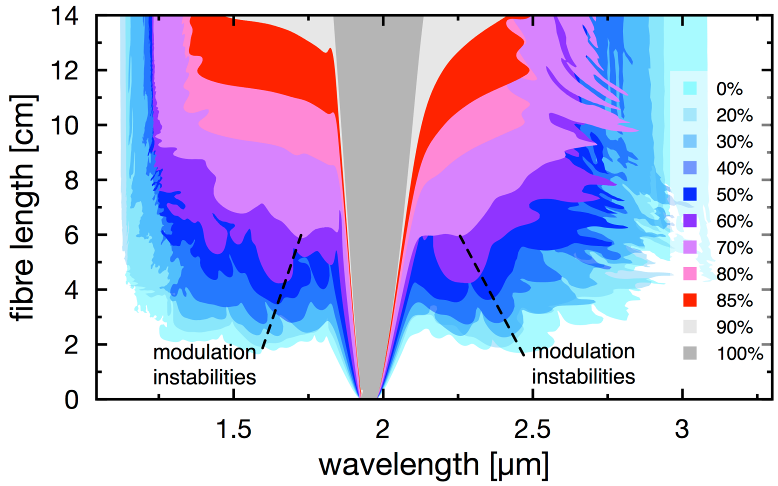

Supplementary Fig. 3: Impact of non-instantaneous nonlinearity on broadening characteristics. *Contour of simulated spectral evolution (at 42 dB contrast to the maximum total spectral intensity) of a 450 fs sech^2 pulse (1 nJ) in a CS_2-core with inner diameter of 4.7 µm for increasing molecular fraction (see legend). The simulations include photon noise to highlight the spectral effects of modulations instabilities, too.*

Supplementary Figure 4 shows the impact of a small change of the molecular contribution to the shot-to-shot spectral stability, i.e. the first order coherence. The spectral coherence drops drastically from f_m = 0.85 to f_m = 0.80, implying a threshold of f_m > 0.80 for the pulse parameters selected here.

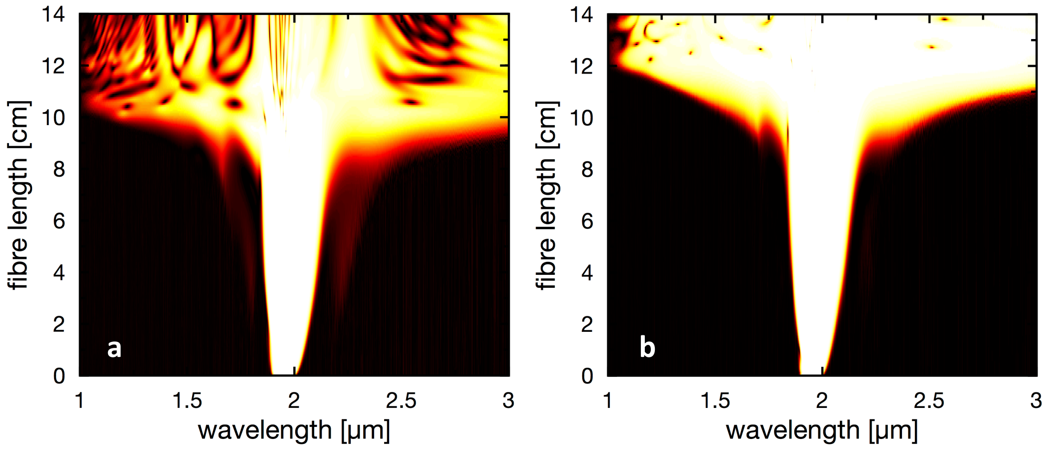

Supplementary Fig. 4: Molecular contribution threshold for perfect coherence. *Evolution of the first-order coherence of a sech² pulse (1 nJ, 450 fs) along a CS_2-core fibre with (a) 80% and (b) 85% molecular contribution to the total nonlinearity. The colorscale ranges from 0 (black, no coherence) to 1 (white, perfect coherence).*

Supplementary Note 3: Liquid-core Fibre Properties

Suppl. Fig. 5 shows the waveguide properties of each of the seven transversal modes supported in a CS_2/silica step-index fibre with 4.7 µm core diameter. The V parameter is a measure of the guidance with V = 1 being the empiric limit where lossless propagation can be assumed.

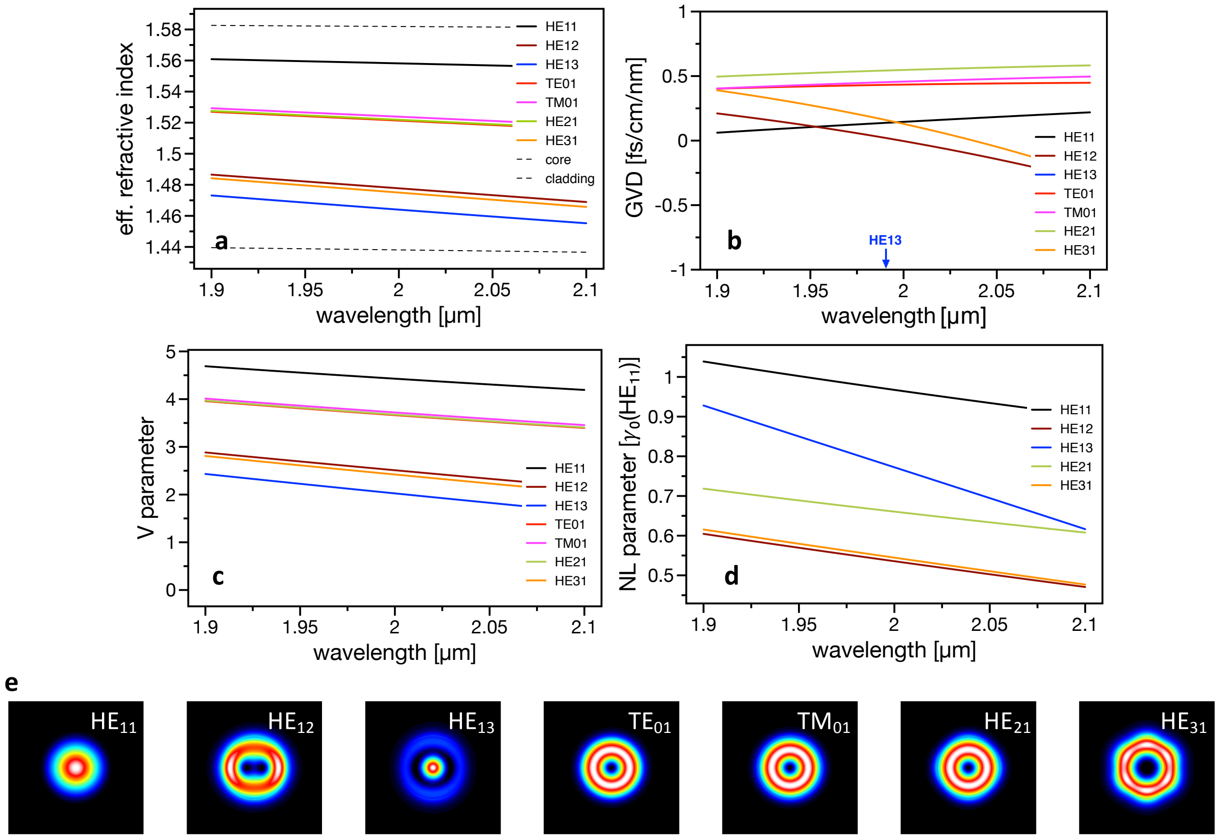

Supplementary Fig. 5: *Optical properties of all modes of a CS_2/silica step-index fibre with 4.7 µm inner diameter: (a) effective refractive index, (b) group velocity dispersion, (c) V parameter, (d) relative nonlinear parameter, (e) intensity distribution.*

Supplementary Note 4: Ultrafast Laser System and Transmission Limits

In the experiment a table-top fibre laser with two amplification stages is used similar to that described in [7] (Suppl. Fig. 6a). The thulium-doped fibres are pumped at 790 nm and the amplification fibres are constantly water cooled at a temperature of 20 °C. A grating compressor is used to compensate the second-order (β_2) phase of the output pulses. An acousto-optical modulator allows step-wise reduction of the pulse repetition rate starting at 11.6 MHz. The system features an output spectrum with a 20 dB bandwidth of 26 nm (Suppl. Fig. 6b) and near-transform-limited optical pulses with a FWHM width of 460 fs (Suppl. Fig. 6c). The pulse shape was reconstructed from the Fourier transform of the output spectrum and a third-order phase offset (β_3 = -0.025 ps^3) was added to match the recorded auto-correlation.

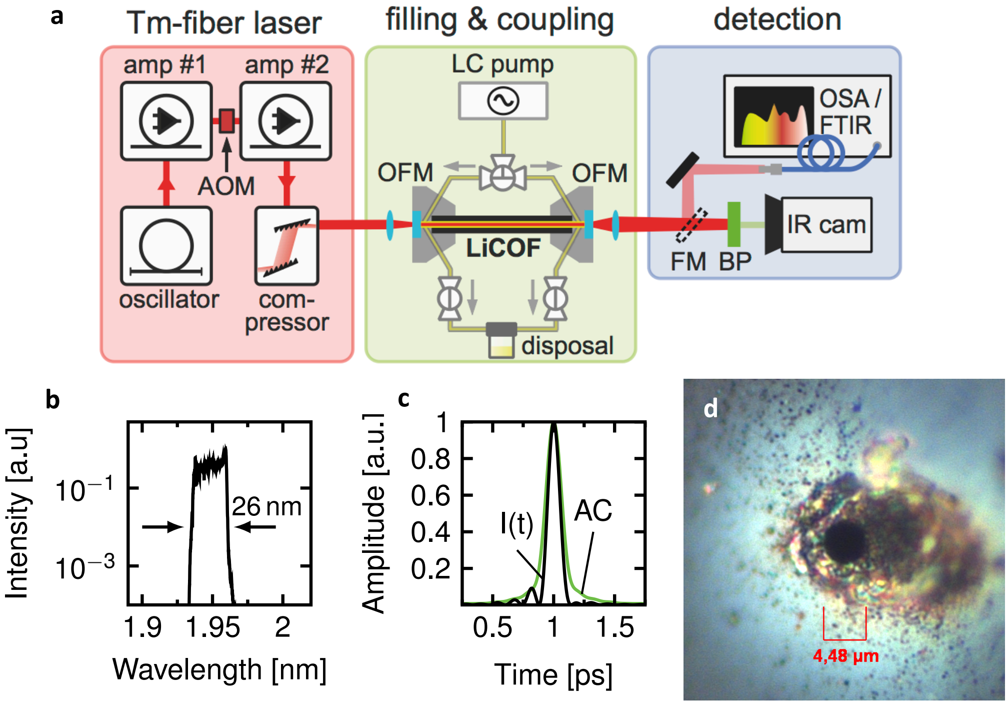

Supplementary Fig. 6: Experimental details. (a) Scheme of the experimental setup. (b) *Output spectrum and (c) pulse shape I(t) reconstructed from the measured autocorrelation (AC) and spectrum of the Tm-based laser source. (d) Microscope image of the capillary input after transmission drop was measured (average power limit). A yellowish residue around the capillary hole is visible.*

Supplementary Table 1 shows the experimental damage thresholds of the liquid-core fibre indicated by a sudden transmission drop observed for various input pulse parameters and pulse repetition rates ν_{rep}. The pulse peak intensity I_0 is calculated in the focus assuming 50 % coupling efficiencies (as measured for low power coupling). Comparing the three parameter sets, we find that there are two origins for the drop: (a) thermal load for high average power (Suppl. Tab. 1, row 3, i.e., high repetition rate, thus low pulse energy), and (b) for high pulse energy (Suppl. Tab. 1, row 1, i.e., low repetition rate, low average power). In case (a) a yellow precipitation debris on the input side was observed (Suppl. Fig. 6d), indicating dissociation of the CS_2 molecules. In case (b) instead, no visible damage or residue could be found at the input or along the capillary. Another measurement at intermediate repetition rate (Suppl. Tab. 1, row 2) shows a transmission drop in the vicinity of both high peak power and high average power with values being in significantly good agreement to the individual power limits measured at lower or higher repetition rate, respectively.

ν_{rep} [MHz]	P_{ave} [mW]	E_p [nJ]	P_0 [kW]	I_0 [TW/cm^2]
2.50	80.0	27.7	52.5	0.882
5.62	152.2	27.1	51.3	1.295
11.24	>152.2	13.6	26.9	0.649

Supplementary Tab. 1: *Damage thresholds of the CS_2/silica fibre. Input pulse parameters when a transmission drop was detected for three individual LiCOF samples with core diameters between 4.4 and 4.8 µm.* ν_{rep}*: pulse repetition rate;* P_{ave}*: average power;* E_p*: pulse energy;* P_0*: pulse peak power;* I_0*: pulse peak intensity. Underlined quantities mark common values between the measurements.*

Supplementary Figure 7 shows the power characteristic of the liquid-core fibre used in the supercontinuum experiments, i.e. input versus output pulse energy. It correlates well with the our simulation results revealing two domains of linear loss. The slope of the curve changes at the supercontinuum onset energy (E_p < 2.5 nJ), after which the broadened spectra approach the strong absorption at 2.25 µm. The slope difference between experiment and simulation in the second domain originate from inaccuracies of our fit model for the CS_2 absorption, which cannot be improved on the basis of the old absorption data. However, the data reveal that the absorption of the system is known and dominantly linear.

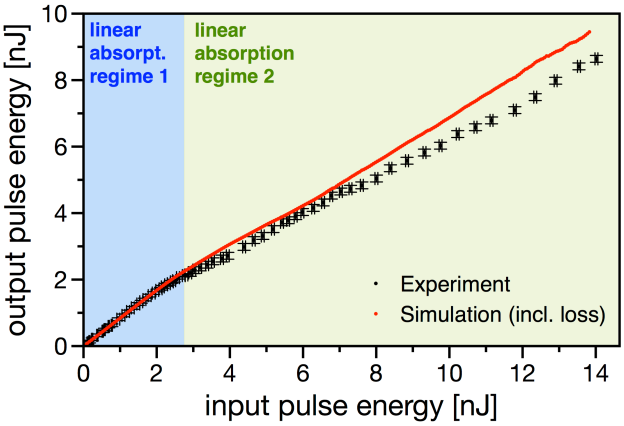

Supplementary Fig. 7*: Comparison of the input/output power characteristic of our system between experiment and simulation. Two linear absorption regimes are distinguishable by two different slopes of the linear power characteristics. Nonlinear absorption does not appear to be dominant.*

Supplementary References

[1] Pfund, a. H. (1935). The Dispersion of CS2 and CCL4 in the Infrared. *Journal of the Optical Society of America*, *25*(11), 351.

[2] Wilhelmi, B. (1967). Über die Anwendung von Dispersionsrelationen zur Bestimmung optischer Konstanten. *Annalen Der Physik*, *474*(5-6), 244. doi:10.1002/andp.19674740504

[3] Ghosal, S., Ebert, J. L., & Self, S. A. (1993). The infrared refractive indices of CHBr3, CCl4 and CS2. *Infrared Physics*. doi:10.1016/0020-0891(93)90120-V

[4] Samoc, A. (2003). Dispersion of refractive properties of solvents: Chloroform, toluene, benzene, and carbon disulfide in ultraviolet, visible, and near-infrared. *Journal of Applied Physics*, *94*(9), 6167. doi:10.1063/1.1615294

[5] Kedenburg, S., Vieweg, M., Gissibl, T., & Giessen, H. (2012). Linear refractive index and absorption measurements of nonlinear optical liquids in the visible and near-infrared spectral region. *Optical Materials Express*, *2*(11), 1588. doi:10.1364/OME.2.001588

[6] Reichert, M., Hu H., Ferdinandus, M. R., Seidel, M., Zhao, P., Ensley, T. R., Peceli, D., Reed, J. M., Fishman, D. A., Webster, S., Hagan, D. J., and Van Stryland, E. W. (2016). Temporal, spectral, and polarization dependence of the nonlinear optical response of carbon disulfide: erratum. Optica 3(6), 657. doi: 10.1364/OPTICA.3.000657

[7] Gebhardt, M., Gaida, C., Hädrich, S., Stutzki, F., Jauregui, C., Limpert, J., & Tünnermann, A. (2015). Nonlinear compression of an ultrashort-pulse thulium-based fiber laser to sub-70 fs in Kagome photonic crystal fiber. *Optics Letters*, *40*(12), 2770. doi:10.1364/OL.40.002770
